# Reviewing South Africa’s malaria elimination strategy (2012–2018): progress, challenges and priorities

**DOI:** 10.1186/s12936-016-1497-x

**Published:** 2016-08-27

**Authors:** Jaishree Raman, Natashia Morris, John Frean, Basil Brooke, Lucille Blumberg, Philip Kruger, Aaron Mabusa, Eric Raswiswi, Bridget Shandukani, Eunice Misani, Mary-Anne Groepe, Devanand Moonasar

**Affiliations:** 1 Centre for Opportunistic, Tropical and Hospital Infections, National Institute for Communicable Diseases, Johannesburg, South Africa; 2 Wits Research Institute for Malaria, University of Witwatersrand, Johannesburg, South Africa; 3 Institute for Sustainable Malaria Control, University of Pretoria, Pretoria, South Africa; 4 Health GIS Centre, South African Medical Research Council, Durban, South Africa; 5 Division of Public Health Surveillance and Response, National Institute for Communicable Diseases, Johannesburg, South Africa; 6 Department of Health and Social Welfare, Limpopo Provincial Government, Tzaneen, South Africa; 7 Department of Health and Social Services, Mpumalanga Provincial Government, Nelspruit, South Africa; 8 Department of Health KwaZulu-Natal, KwaZulu-Natal Provincial Government, Jozini, South Africa; 9 Malaria Directorate, National Department of Health, Pretoria, South Africa; 10 WHO Intercountry Support Team, Pretoria, South Africa

**Keywords:** Malaria, Elimination, South Africa, Vector control, Case management, Surveillance, Challenges, Priorities

## Abstract

**Background:**

With a sustained national malaria incidence of fewer than one case per 1000 population at risk, in 2012 South Africa officially transitioned from controlling malaria to the ambitious goal of eliminating malaria within its borders by 2018. This review assesses the progress made in the 3 years since programme re-orientation while highlighting challenges and suggesting priorities for moving the malaria programme towards elimination.

**Methods:**

National malaria case data and annual spray coverage data from 2010 until 2014 were assessed for trends. Information on surveillance, monitoring and evaluation systems, human and infrastructure needs and community malaria knowledge was sourced from the national programme mid-term review.

**Results:**

Malaria cases increased markedly from 6811 in 2013 to 11,711 in 2014, with Mpumalanga and Limpopo provinces most affected. Enhanced local transmission appeared to drive malaria transmission in Limpopo Province, while imported malaria cases accounted for the majority of cases reported in Mpumalanga Province. Despite these increases only Vhembe and Mopani districts in Limpopo Province reported malaria incidences more than one case per 1000 population at risk by 2014. Over the review period annual spray coverage did not reach the recommended target of 90 % coverage, with information gaps identified in parasite prevalence, artemether-lumefantrine therapeutic utilization, asymptomatic/sub-patent carriage, drug efficacy, vector distribution and insecticide resistance.

**Conclusions:**

Although South Africa has made steady progress since adopting an elimination agenda, a number of challenges have been identified. The heterogeneity of malaria transmission suggests interventions in Vhembe and Mopani districts should focus on control, while in KwaZulu-Natal Province eliminating transmission foci should be prioritized. Cross-border initiatives with neighbouring countries should be established/strengthened as a matter of urgency since malaria importation poses a real threat to the country’s elimination efforts. It is also critical that provincial programmes are adequately resourced to effectively conduct the necessary targeted elimination activities, informed by current vector/parasite distribution and resistance data. More sensitive methods to detect sub-patent infections, primaquine as a transmission-blocking drug, and alternative vector control methods need to be investigated. Knowledge gaps among malaria health workers and affected communities should be identified and addressed.

## Background

**Fig. 1:**
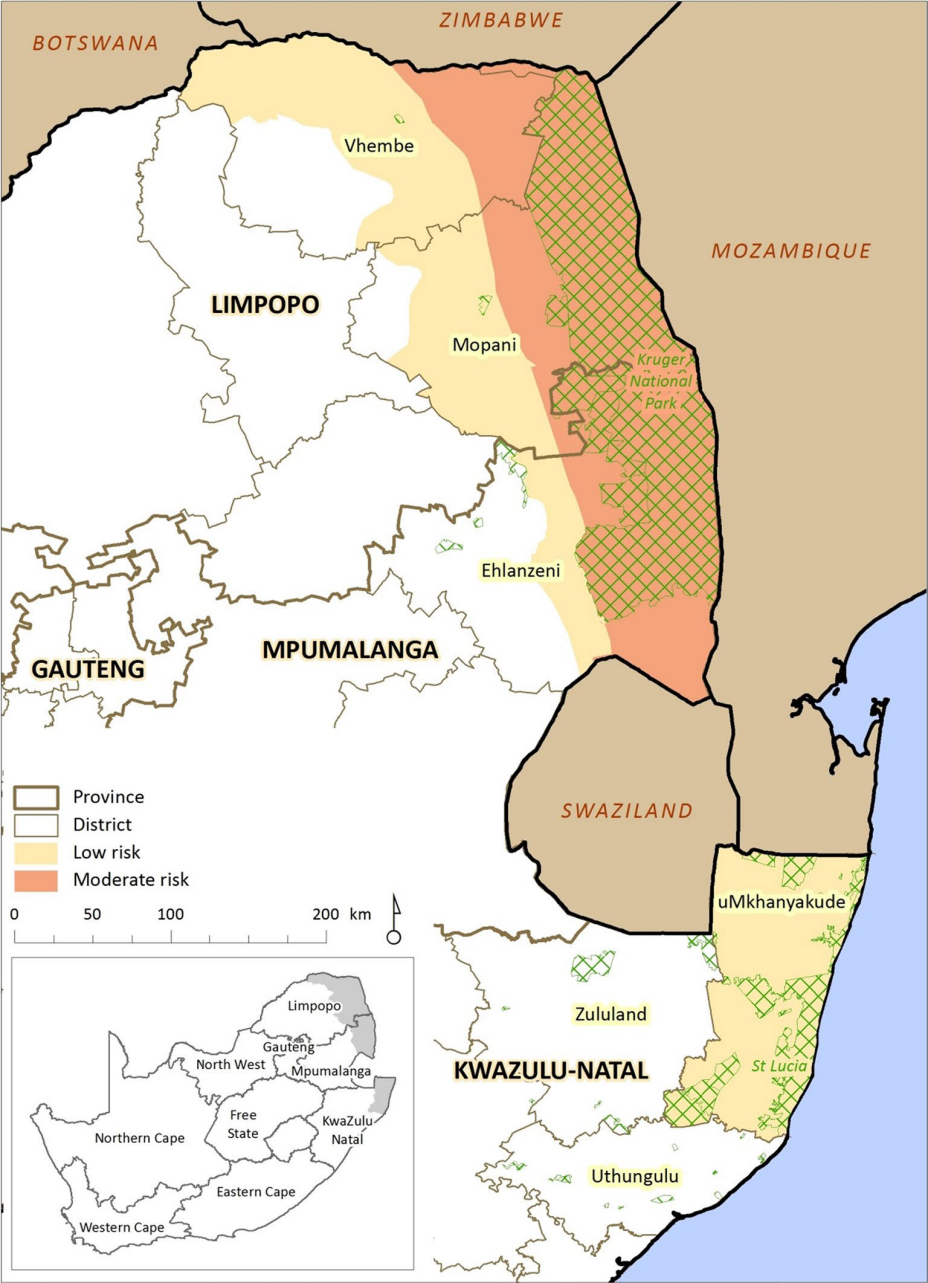
Malaria risk map for South Africa (*Source* South African Medical Research Council). Cross-hatched areas are game reserves

Malaria is endemic to only three of South Africa’s nine provinces and is currently restricted to the low altitude border regions of these three provinces (Fig. 1) [1]. A robust malaria vector control and surveillance strategy dating back to the early 1940s ensured decades of effective malaria control resulting in the near elimination of malaria in South Africa by 1970 [2]. Unfortunately, favourable climatic factors (high rainfall and flooding) resulted in malaria resurging during the 1972/1973 malaria season. Heightened surveillance, prompt treatment with chloroquine together with dichloro-diphenyl-trichloroethane (DDT)-based indoor residual spray (IRS) operations brought malaria back under control until the mid-1980s [2].

The establishment of chloroquine-resistant parasites in the region caused a minor spike in malaria cases during the mid- to late-1980s, which was rapidly reversed by the replacement of chloroquine with sulfadoxine-pyrimethamine as first-line treatment [3]. Malaria remained under control until the mid-1990s when malaria case numbers began increasing once again, culminating in the country’s worst malaria epidemic to date, with over 60,000 reported cases at the height of the outbreak in 2000 [4]. Favourable climatic factors (elevated temperatures and rainfall), increased population movement across the country’s borders, together with the increased prevalence of drug-resistant parasites and insecticide-resistant vectors, particularly *Anopheles funestus* [5], have been identified as major contributing factors [6].

Since the 1999/2000 malaria outbreak, enhanced cross-border malaria control [7], the introduction of artemisinin-based combination therapy (ACT) for the treatment of uncomplicated malaria [8] and the re-introduction of DDT for IRS operations [9] have contributed to a marked and sustained decline in malaria prevalence. With fewer than 6000 cases notified in 2007 [10], the Malaria Directorate of the South African Department of Health initiated an internal dialogue on malaria elimination in accordance with the World Health Organization (WHO) recommendations [11]. These deliberations concluded with South Africa formally adopting an elimination strategy in 2012, aimed at halting local malaria transmission within the country’s borders by 2018 [12]. The elimination strategy consists of four clearly-defined key objectives, namely:

- To strengthen passive and active surveillance and monitoring and evaluation systems so that *100 %* of districts report promptly and routinely on key malaria indicators by *2015*
- To ensure that all levels of the malaria programme have *sufficient capacity* to coordinate and implement malaria interventions by *2016*
- To ensure that *100 %* of the population has adequate knowledge, attitudes and practices on malaria by *2018* through appropriate information, education and communication (IEC), social mobilization and advocacy
- To effectively prevent malaria infections and *eliminate all parasite reservoirs* in South Africa by *2018*.

Embedded within these broad objectives are three critical district level milestones, namely:

- Three districts in Limpopo (Capricorn, Waterberg, and Sekhukhune) and two districts in KwaZulu-Natal (Zululand and uThungulu) with <0.1 case per 1000 population at risk in 2012, reaching zero local cases by 2014
- Three additional districts within the three malaria endemic provinces achieving zero local cases by 2016, and finally,
- All malaria endemic districts in South Africa reporting zero local cases by 2018.

A mid-term review of the national malaria elimination strategic plan was conducted in July 2015 to assess progress made toward the successful completion of these critical milestones [13].

This paper reviews the progress made in the 3 years since South Africa’s official transition from malaria control to malaria elimination. It highlights the technical, policy and operational challenges that the country faces in achieving its elimination milestones while identifying opportunities that must be leveraged to ensure South Africa’s 2018 goal of malaria elimination becomes a reality.

## Methods

### Country setting

Malaria is seasonal in South Africa, occurring between September and May with cases generally peaking after the Christmas and Easter holidays. Over 90 % of the reported cases are a result of *Plasmodium falciparum* infections with *An. arabiensis*, the most likely mosquito vector [14]. At present malaria is restricted to the low-altitude border regions of KwaZulu-Natal, Mpumalanga and Limpopo with approximately 10 % of the country’s population residing within a malaria risk area.

Within KwaZulu-Natal three municipal districts, uMkhanyakude, uThungulu and Zululand, are currently classified as malaria endemic. In the recent past imported malaria has accounted for the majority of the cases reported from these three districts, placing them within the elimination phase of the WHO elimination continuum [6]. Of the three districts in Mpumalanga, namely Ehlanzeni, Gert Sibande, and Nkangala, at present only Ehlanzeni is classed as malaria endemic. Despite being the major contributor to Mpumalanga’s malaria burden, Ehlanzeni is considered to be in the elimination phase of the WHO elimination continuum as most of the cases reported in the district are imported [6]. In Limpopo, there are five malaria endemic municipal districts, namely Capricorn, Greater Sekhukhune, Mopani, Vhembe and Waterberg. Vhembe contributed to more than 60 % of the national malaria burden and is considered to be in the control phase of the elimination continuum, with the remaining districts either in the elimination or prevention of reintroduction phase [6].

In accordance with the national malaria treatment guidelines [15], all fever cases presenting at health facilities must be tested for malaria by *P. falciparum* specific rapid diagnostic test (RDT) or microscopy. Uncomplicated falciparum infections are treated with artemether-lumefantrine (Coartem^®^) while either IV quinine or IV artesunate are used to treat complicated malaria cases.

### Morbidity and mortality data

Health-care workers at health facilities within the three malaria endemic provinces enter confirmed malaria case data into clinic or hospital registers as well as report cases to the district health office telephonically. In addition individual malaria case records are entered onto malaria notification forms, which are forwarded weekly to the provincial malaria control programmes (MCP). At the MCP offices individual patient demographic and case management data are captured onto a computerized malaria information system (MIS) [16]. A case report is then generated by the MIS, which is issued to the surveillance agent or malaria case investigator for follow-up and investigation. Once the follow-up investigation is completed the completed forms are returned to MCP offices, where any new information pertaining to the case is entered into the MIS using the patient’s unique identifier, thereby ensuring the new data are linked to the original patient record. Individual and aggregate malaria case data from each provincial MIS are transferred on a monthly basis to a national integrated MIS developed by the South African Medical Research Council and housed at the National Institute for Communicable Diseases (NICD) in Johannesburg, South Africa.

### Malaria case definition

All confirmed malaria cases are classified as either local or imported [17]. A local (autochthonous) case is defined as a malaria infection acquired within a malaria-receptive area of South Africa where there is no history of travel to another malaria endemic country and where local transmission cannot be disproven. An imported malaria case is defined as an infection whose origin can be traced to a known malarious area outside of South Africa to which the individual has travelled. In instances where local transmission is unlikely but the malaria patient cannot be traced to verify travel history, the case is categorized as unclassified.

### Indoor residual spraying

Currently in South Africa generalized IRS operations are conducted in the malaria-affected areas of the three malaria-endemic provinces, using a mosaic strategy comprising pyrethroids and DDT and, in certain instances carbamates, irrespective of malaria incidence. At the beginning of each malaria season provincial MCPs determine the number of structures to be sprayed, guided by the number of structures within the malaria endemic area, availability of insecticide and available insecticide resistance data. Spray personnel record the number of rooms and structures sprayed with insecticide on spray cards. The spray card data is verified by an IRS team leader and then submitted to the provincial MCP where the spray data are entered into a spraying information system (SIS). The SIS allows for the rigorous monitoring and evaluation of IRS operations.

### Mid-term review

Data on the surveillance, monitoring and evaluations systems, programme human and infrastructure capacity, as well as community knowledge of malaria, were sourced from the report produced following the mid-term review of the national malaria elimination strategy [13].

### Statistical analysis

Descriptive statistical analysis was conducted for all variables identified as important. Statistical significance was set at 5 % with data analysis carried out using Stata version 13.1 (State Cooperation, College Station, TX, USA).

## Results

### National malaria-related morbidity and mortality data

**Fig. 2:**
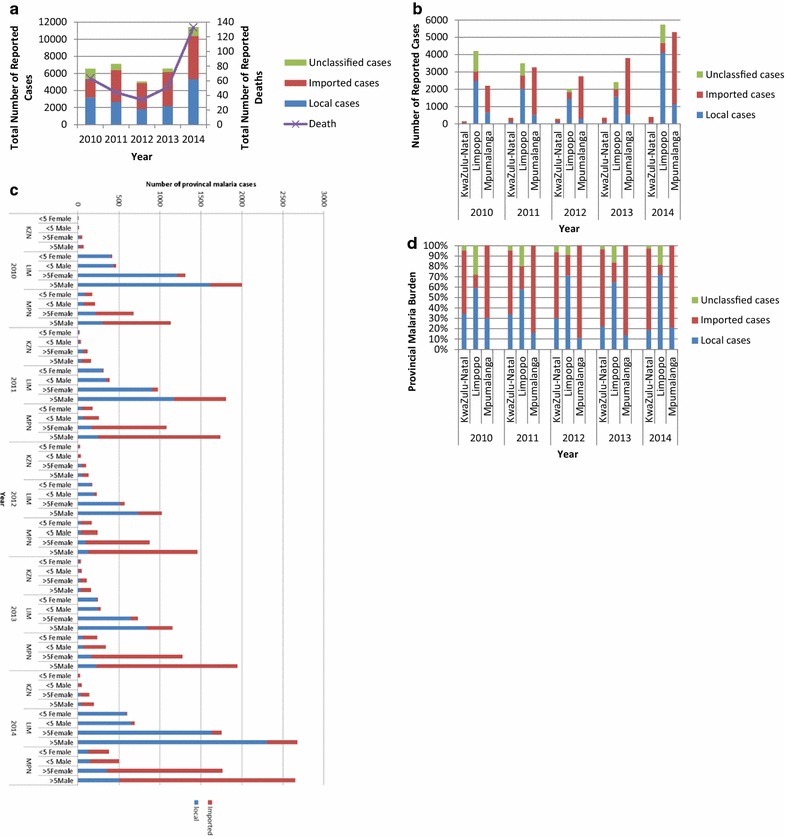
**a** Annual number of malaria cases (local, imported and unclassified) and deaths in South Africa between 2010 and 2014. **b** Annual number of malaria cases (local, imported and unclassified) by province in South Africa between 2010 and 2014. **c** Annual number of malaria cases (local and imported) in under- and over-5-year-old individuals by gender and province in South Africa between 2010 and 2014. *KZN* KwaZulu-Natal, *LIM* Limpopo, *MPN* Mpumalanga. **d** Contribution of local, imported and untraceable malaria cases to each province during the period 2010–2014

Although malaria case numbers increased marginally from 6548 in 2010 and to 7104 in 2011, they declined markedly to 5065 in 2012 (Fig. 2a). Unfortunately since 2012 the number of reported malaria cases has increased annually, peaking at 11,432 in 2014. Over the review period malaria case numbers from all three malaria endemic provinces mirrored the national trend (Fig. 2b) with KwaZulu-Natal consistently contributing the least to the national malaria burden. The major contributor to the national malaria burden from the endemic provinces alternated between Limpopo and Mpumalanga, with Limpopo accounting for majority of the cases reported in 2014 (Fig. 2b).

Between 2010 and 2012 national malaria-related deaths almost halved from 63 in 2010 to 34 in 2012, but have subsequently increased annually, mirroring the trend in malaria cases (Fig. 2a). Over the review period malaria deaths increased significantly from 63 in 2010 to 133 by 2014 (OR 1.09; 95 % CI 1.01–1.17; p = 0.024; Fig. 2a). Univariate analysis revealed that while females were marginally more likely to contract malaria (OR 1.02; 95 % CI 1.00–1.03; p = 0.038; Fig. 2c), pregnancy did not increase the risk of infection (OR 0.74; 95 % CI 0.63–0.86; p < 0.0001). The odds of children under the age of 5 years contracting malaria remained unchanged during the study period (OR 1.01; 95 % CI 0.99–1.03; p = 0.286; Fig. 2c).

**Fig. 3:**
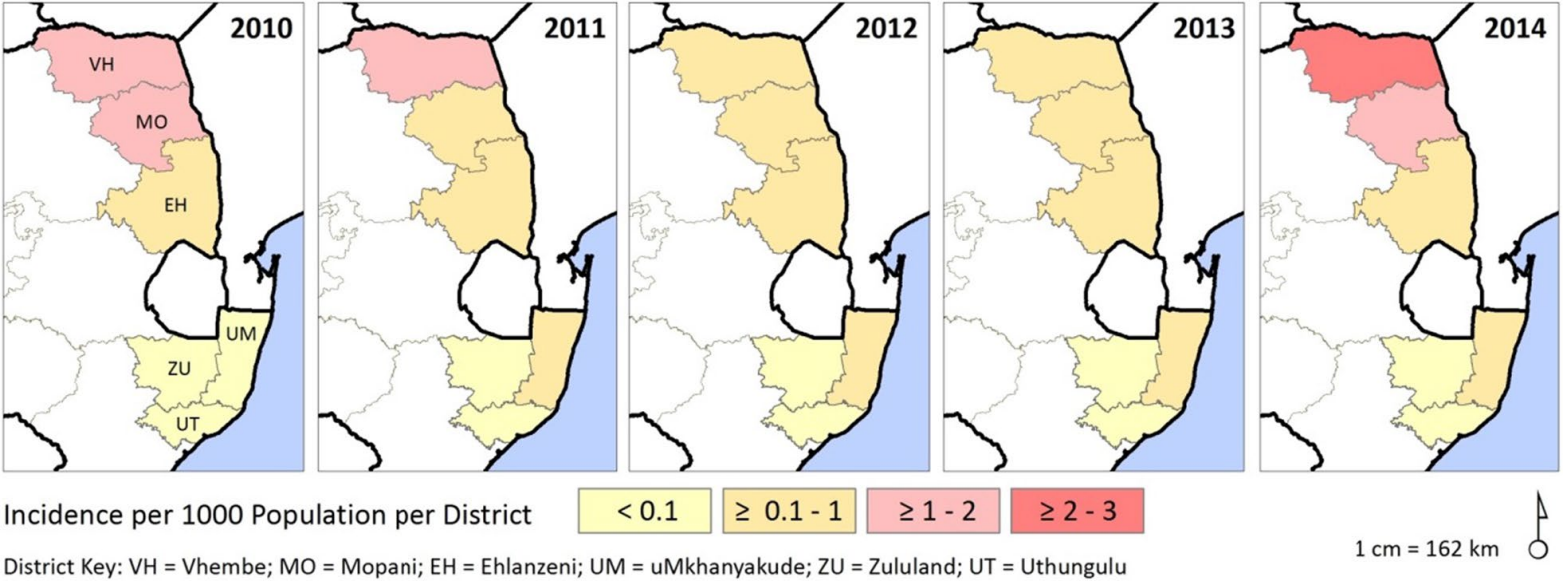
Malaria incidence in South Africa by municipal district for the period 2010–2014

A closer inspection of the morbidity and mortality data revealed that the major source of the malaria infection varied greatly between the provinces (Fig. 2c, d). Local cases accounted for approximately 35 % of the cases reported in KwaZulu-Natal in 2010, and this value declined significantly to 18 % by 2014 (OR 0.79; 95 % CI 0.74–0.84; p < 0.0001; Fig. 2c, d). In contrast local cases accounted for the majority of Limpopo’s malaria burden, increasing significantly from 58 % in 2010 to 71 % in 2014 (OR 1.16; 95 % CI 1.14–1.18; p < 0.0001; Fig. 3). While the contribution of local cases to Mpumalanga’s malaria burden declined significantly over the study period (OR 0.94; 95 % CI 0.92–0.97; p < 0.0001; Fig. 2d), an increase in local cases from 12 to 21 % between 2012 and 2014 was noted (Fig. 2c, d).

### Provincial malaria morbidity and mortality data

#### KwaZulu-Natal Province

Over the past 5 years KwaZulu-Natal Province has consistently contributed to <1 % of the South Africa’s national malaria burden. During the review period, the provincial district of uMkhanyakude notified the highest number of malaria cases (over 95 %) with Zululand reporting the least. By 2014 malaria incidence in uMkhanyakude, uThungulu and Zululand districts was 0.10, 0.02 and 0.01 per 1000 population at risk, respectively (Fig. 3).

The number of imported cases increased significantly across three endemic provincial districts from 92 in 2010 to 320 in 2014 (OR 1.27; 95 % CI 1.19–1.35; p < 0.0001), with females less frequently associated with imported malaria cases (OR 0.62; 95 % CI 0.51–0.75; p < 0.0001; Fig. 2c). Although malaria-related mortality remained unchanged over the study period (OR 0.76; 95 % CI 0.54–1.07; p = 0.113), individuals with locally-acquired malaria infections were four times less likely to survive compared to individuals with imported malaria (OR 4.14; 95 % CI 1.43–11.98; p = 0.009). Children under 5 years of age were more likely to acquire a local malaria infection compared to all other age groups (OR 1.36; 95 % CI 1.06–1.74; p = 0.017; Fig. 2c).

#### Limpopo Province

In contrast to KwaZulu-Natal, Limpopo Province has contributed significantly to South Africa’s malaria burden, accounting for most of the reported cases in 2010, 2011 and 2014 (Fig. 2b). Of the five provincial malaria endemic districts, Vhembe is the highest burdened district, followed by Mopani, with the remaining three districts mainly reporting imported cases. By 2014 malaria incidence in Vhembe was 2.4 and 1.7 in Mopani (Fig. 3).

The number of locally-acquired cases increased significantly over the study period from 2478 in 2010 to 4116 in 2014 (OR 1.16; 95 % CI 1.14–1.18; p < 0.0001). Although unclassified cases decreased markedly from 1175 in 2010 to 181 in 2012, they began increasing thereafter, reaching 1053 by 2014. Despite this sharp increase in unclassified cases since 2012, the odds of a case remaining unclassified declined significantly over the study period (OR 0.88; 95 % CI 0.86–0.90; p < 0.0001). As seen in KwaZulu-Natal, females (OR 1.84; 95 % CI 1.73–1.97; p < 0.0001; Fig. 2c) and children under the age of five (OR 1.80; 95 % CI 1.66–1.95; p < 0.0001; Fig. 2c) in Limpopo Province were more likely to locally acquire malaria infections. Individuals who acquired an infection locally were two times less likely to survive than individuals who acquired the infection outside of South Africa (OR 2.35; 95 % CI 1.65–3.34; p < 0.0001).

#### Mpumalanga Province

While Mpumalanga Province, like Limpopo Province, contributes significantly to South Africa’s malaria burden, the majority of the cases reported in Mpumalanga Province are classified as imported with almost all cases notified in the district of Ehlanzeni. The number of imported cases increased over the study period from 1530 in 2010 to 4168 in 2014 (OR 1.06; 95 % CI 1.03–1.08; p < 0.0001). Once again females (OR 1.21; 95 % CI 1.11–1.30; p < 0.0001; Fig. 2c) and children under the age of five (OR 1.88; 95 % CI 1.71–2.07; p < 0.0001; Fig. 2c) were more likely to acquire malaria infections locally. Individuals who acquired an infection within Mpumalanga Province were three times less likely to survive the malaria infection compared to those infected with malaria outside of South Africa (OR 3.40; 95 % CI 2.33–4.97; p < 0.0001).

### Imported cases

Of the 36,712 cases reported over the review period 17,511 (47.6 %) were classified as imported cases. Most of the imported cases originate from other African countries (Table 1) with the occasional case imported from Asia (data not shown). Mozambique accounted for 87.3 % (15,287/17,511) of all imported cases followed by Zimbabwe (5.8 %, 1025/17,511) (Table 1).

**Table 1:** Ten most common African source countries of imported malaria reported in South Africa for the period 2010–2014

Country	Number of cases
Mozambique	15,287
Zimbabwe	1025
Ethiopia	403
Somalia	304
Swaziland	130
Malawi	110
Zambia	44
Democratic Republic of Congo	35
Tanzania	27
Congo	18

### Spray coverage

Both Mpumalanga and Limpopo provinces achieved an average annual IRS coverage of 85 % and above (Table 2) during the review period. Although KwaZulu-Natal generally achieved a spray coverage of above 80 %, only 69 % of the targeted structures were sprayed during IRS operations in 2012 (Table 2).

**Table 2:** Number of structures sprayed and spray coverage achieved in South Africa by province from 2010 until 2014

Year	Province	Structures targeted	Structures sprayed	Spray coverage (%)
2010	KwaZulu-Natal	287,639	263,819	91.7
	Limpopo	1,080,859	967,502	89.5
	Mpumalanga	699,105	653,007	93.4
2011	KwaZulu-Natal	265,771	238,829	89.9
	Limpopo	1,226,731	1,064,396	86.8
	Mpumalanga	563,587	519,500	92.2
2012	KwaZulu-Natal	324,690	224,036	69.0
	Limpopo	1,295,671	1,140,588	88.0
	Mpumalanga	537,862	507,226	94.3
2013	KwaZulu-Natal	309,004	256,335	83.0
	Limpopo	1,290,513	1,104,446	85.6
	Mpumalanga	490,396	448,468	91.5
2014	KwaZulu-Natal	322,010	290,505	90.2
	Limpopo	1,501,929	1,298,445	86.5
	Mpumalanga	696,264	632,574	90.9

### Surveillance, monitoring and evaluation systems

#### Case reporting

While most malaria cases detected at primary health care facilities were reported to the district and provincial malaria offices, this reporting generally did not take place within the required 24 h. These delays in case notification impeded both prompt reactive case investigations and the monitoring of malaria case data in real time at the provincial and national levels.

In an attempt to improve 24-h case reporting, the National Malaria Directorate together with the Clinton Health Access Initiative developed a cellular application, MalariaConnect, which allows for immediate case reporting using cellular devices at no cost to the end user. Since the phased roll-out of the application commenced in August 2015, 305 facilities across five districts (Vhembe, Mopani, Ehlanzeni, uMkhanyakude and uThungulu) within the three endemic provinces have received training on the MalariaConnect application. Although all 305 facilities have begun using MalariaConnect, only 62 % of all confirmed cases at these facilities were being reported through MalariaConnect. Encouragingly however, 85 % of all cases reported using MalariaConnect was notified within 24 h. This has resulted in a marked improvement in case investigation response time, from an average of 6 days to an average of 3 days.

Currently each malaria endemic province has its own MIS where all malaria case data are captured. Although data collected by these three information systems are not uniform, a core set of essential data variables are maintained and captured by all three systems. Captured provincial case data are transferred to the national integrated MIS on a regular basis using a web-based platform.

#### Epidemic preparedness and response systems

As an interim measure, while a national malaria early warning system (MEWS) is being developed, the national malaria control programme established epidemic thresholds based on 5 years of retrospective national case data to support provincial elimination efforts. In addition, certain provincial control programmes developed their own epidemic thresholds using retrospective provincial case data. While provincial response plans in the event of a threshold being breached are in place, financing of these responses remains a challenge.

#### Entomological and insecticide resistance monitoring

Varying levels of entomological surveillance and insecticide resistance monitoring are being undertaken in all three provinces, depending on available resources (human, infrastructural and financial). The NICD has assisted both Mpumalanga and KwaZulu-Natal with routine entomological surveillance and insecticide resistance monitoring. Detailed information on insecticide susceptibility status by vector species and province can be found in Brooke et al. [14, 18].

### Human and financial resource capacity

Both the national and provincial MCP are experiencing a severe shortage of technical experts at all levels. This lack of capacity continues to impact negatively on every facet of the elimination programme. The limited funds available for effective implementation of the highly resource-dependent elimination agenda is placing increased strain on already financially over-stretched provincial control programmes. The Malaria Directorate has attempted to garner funds from external sources but thus far funds raised have been insufficient to fill the identified resource gaps.

## Discussion

In the 3 years since adopting the elimination agenda, South Africa has taken some positive steps towards achieving its 2018 elimination target. The potential for onward transmission has been significantly reduced by the increased access to point-of-care malaria diagnostics, prompt reactive case investigations facilitated through the implementation of a 24-h case reporting system, and improved surveillance for vectors and insecticide resistance. In addition the collection of more detailed travel and behavioural data during case investigations has enabled more rigorous case verification and more accurate case classification. This improved case classification helped inform appropriate intervention implementation while providing an indication of progress towards elimination. Notwithstanding these advancements, numerous challenges have been identified, which have the potential to jeopardize South Africa’s elimination goals if not adequately addressed.

Like most of the malaria endemic countries within southern Africa, South Africa experienced an upsurge in malaria cases in 2014 [19]. This regional increase appears to have been driven primarily by favourable climatic conditions (optimum rainfall and ideal temperatures). Encouragingly, despite the regional increase in 2014, 10 of the 12 South African malaria-endemic districts maintained a malaria incidence of <1 case per 1000 population at risk. The two districts, Vhembe and Mopani, where the WHO elimination threshold [11] was exceeded, are located in Limpopo Province, the highest-burdened malaria-endemic province.

Vhembe has been, and continues to be, the most affected malaria district in South Africa, accounting for over 60 % of all cases reported annually. As malaria transmission intensity decreases, malaria incidence becomes more heterogeneous [11], resulting in a stratification of intervention needs. The elevated malaria incidence in both Vhembe and Mopani districts implies a need for the implementation of enhanced integrated generalized control measures in which options for controlling malaria importation are explored.

In total contrast to Limpopo Province, all three malaria endemic districts in KwaZulu-Natal had reached the minimum elimination threshold by 2010 [6] and remained there through the review period. Although these districts did not achieve the critical milestone of zero local cases by 2014, the sustained low incidence suggests local elimination is possible if hotspots (areas of persistent residual transmission) are eliminated [20, 21]. Hotspots fuel onward transmission, therefore targeting them reduces transmission intensity, positively impacting both the most affected households and the community as a whole. As optimum coverage of the targeted intervention is essential for hotspot elimination [20], selecting the appropriate combination of interventions targeting the vector and/or parasites is vitally important. To facilitate hotspot elimination and possibly fast-track malaria elimination in KwaZulu-Natal, a needs assessment for hotspot identification and elimination should be prioritized.

Despite contributing significantly to South Africa’s national malaria burden, over 80 % of the cases reported in Mpumalanga are classified as imported [22]. Reactive case-detection data revealed South Africa’s high-burden neighbours, namely Mozambique and Zimbabwe, are the major source of imported malaria. Mathematical models [23] support the view that malaria elimination cannot be realized in the presence of sustained malaria importation [24–26].

In its recently launched global technical strategy (GTS) for malaria [27], the WHO acknowledged the importance of reducing malaria importation in an elimination setting by including a supportive strategy dedicated to cross-border collaborations. In line with the GTS recommendations, South Africa is engaging in the Elimination Eight (E8) regional initiative whose core objective is to strengthen regional collaborations to eliminate malaria in eight participating countries [28]. In addition, building on the successes of the Lubumbo Spatial Development Initiative, a cross-border collaboration between South Africa, Swaziland and Mozambique [7], South Africa is currently engaging with Mozambique and Swaziland on new cross-border initiative, called the Mozambique, South Africa and Swaziland (MOSASWA) malaria cross-border initiative. The overarching goal of MOSASWA is to achieve zero local transmission in Swaziland, South Africa and Maputo Province, Mozambique, by 2020 and pre-elimination status in southern Mozambique (Maputo and Gaza Provinces) by 2025, through harmonized collaborative efforts.

Underpinning all these cross-border initiatives is the need for a surveillance system sensitive enough to track mobile and migrant populations, diagnostics that accurately detect asymptomatic individuals, sub-patent carriers and gametocyte carriers, transmission-blocking anti-malarials, novel vector surveillance and control methods, as well as appropriately skilled personnel. The accurate detection of all malaria carriers (symptomatic and asymptomatic) is a fundamental requirement of an elimination agenda. However, as transmission intensity continues to decline, the commonly used diagnostic tools, light microscopy and RDTs, lack the sensitivity required to detect sub-patent infections [29, 30]. Novel tools such as ultra-sensitive polymerase chain reaction (uPCR) and loop-mediated isothermal amplification (LAMP), which have been shown to be more sensitive in low transmission settings [31–33], need to be assessed for cost-effectiveness in a rural South African setting.

Similar to other countries where malaria transmission intensity has decreased markedly, adults, as opposed to children and pregnant women, bear the higher malaria risk [34–36]. This heightened risk is most likely driven by social, behavioural and/or occupational factors that increase the odds of adults being exposed to malaria-infected vectors [34, 36]. In contrast to previous studies, adult females were slightly more at risk of contracting malaria, particularly if locally transmitted, compared to adult males. The reason for this is unclear but one possible contributing factor could be that adult males who travel for work and/or recreation are acting as asymptomatic parasite reservoirs and are sustaining local transmission [34].

A rather concerning finding was the increased risk of a negative outcome across all age groups if malaria was contracted within South Africa. One possible reason for this is a low index of suspicion among health-care workers and the general public in light of South Africa’s perceived low malaria risk. Malaria awareness campaigns aimed at improving health-seeking behaviours and case management practices of the general public and health-care workers, respectively, should be prioritized.

All malaria endemic countries neighbouring South Africa have implemented a single-dose primaquine policy as a means of reducing onwards transmission [19], in accordance with a WHO recommendation [37]. As this anti-malarial drug is currently not registered in South Africa, this policy cannot be implemented locally at present. The scientific basis of a single-dose primaquine policy for malaria elimination in South Africa needs to be carefully evaluated.

In addition to the maintenance and improvement of current IRS-based vector control interventions, South Africa needs to explore alternate vector control strategies such as larval source management and technologies that target outdoor-resting adult mosquito vectors. Finally, as community and malaria health worker engagement in, and support of, the elimination agenda is fundamental to its success, knowledge gaps, if any, need to be identified and appropriately addressed.

Based on the findings of this review, key operational issues that should be prioritized to further South Africa’s elimination goals are listed below:

1. Implementation of foci of transmission identification and elimination in the three malaria endemic districts of KwaZulu-Natal, as a means of realising the lapsed 2014 milestone of zero local cases in these districts.
2. Maintaining generalized control intervention with blanket coverage in Vhembe and Mopani districts, Limpopo Province.
3. Operationalisation of cross-border initiatives, particularly with Mozambique, to reduce the importation of malaria.
4. Development of an enhanced surveillance system that allows for the tracking of mobile/migrant populations as well as proactive and reactive case detection.
5. Reactive case detection should become routine in all districts where the WHO elimination threshold of <1 case per 1000 population at risk, has been met. Ideally as part of this investigation either a day 3 or 7 follow-up filter-paper blood sample should be collected from all individuals treated for malaria to allow for the assessment of artemether-lumefantrine efficacy.
6. Evaluation of evidence for a single-dose primaquine policy.
7. Assessment of novel techniques capable of detecting sub-patent and gametocyte carriers.
8. Additional vector control measures, especially those targeting out-door resting vectors, need to be evaluated.
9. Entomological surveillance activities, including routine insecticide resistance monitoring, need to be scaled up.
10. Knowledge gaps among the affected communities and malaria health workers must be regularly assessed and addressed. Appropriate messaging that targets high-risk groups need to be developed.

## Conclusions

Despite the marked increase in local malaria case numbers reported in 2014, South Africa has made considerable progress in implementing its elimination agenda. A 24-h malaria reporting system has been implemented in 305 facilities within the malaria endemic regions, enhanced surveillance for vectors and insecticide resistance has commenced and improved case management measures have been implemented. The sustained implementation of effective interventions has decreased transmission intensity causing malaria to become more heterogeneous. This heterogeneity calls for a stratification of interventions implemented. In areas of high transmission intensity, such as Vhembe and Mopani districts, generalized activities focussed at control should continue. In areas nearing elimination, such as KwaZulu-Natal, targeted activities aimed at identifying and eliminating foci of transmission must become a priority.

If South Africa is to eliminate malaria by 2018, surveillance must be enhanced to allow for the timely identification and elimination of foci of transmission, using the appropriate targeted interventions at optimum coverage. In addition, systems to identify and appropriately treat asymptomatic/sub-patent malaria carriers must be implemented, the importation of malaria tackled by strengthening cross-border initiatives, a primaquine policy to reduce the likelihood of onwards transmission should be investigated, anti-malarial drug therapeutic efficacy assessed regularly, malaria awareness campaigns conducted frequently and the supply of chemoprophylaxis for travellers considered. South Africa’s experiences to date emphasize the need for an intensification of parasite and vector surveillance and implementation of a high-resolution MIS that enables active case detection and management as well as the targeting of priority focus areas, in countries attempting to eliminate malaria.

## Abbreviations

ACT: artemisinin-based combination therapy
DDT: dichloro-diphenyl-trichloroethane
GTS: global technical strategy
IEC: information, education and communication
IRS: indoor residual spray
MCP: malaria control programme
NICD: National Institute for Communicable Diseases
RDT: rapid diagnostic test
